# Tumour-derived exosomes or microvesicles: another mechanism of tumour escape from the host immune system?

**DOI:** 10.1038/sj.bjc.6602360

**Published:** 2005-01-25

**Authors:** T L Whiteside

**Affiliations:** 1 University of Pittsburgh Cancer Institute, Pittsburgh, PA, USA

Tumours are known to be able to successfully escape the host immune system by one or both of the two general mechanisms: a loss of surface antigens to become invisible to immune cells or an attack on immune cells to disable their antitumour functions. Both phenomena are now recognised as significant contributors to cancer growth and disease progression (Whiteside, 2002). While the concept of surface antigen loss was recognised many years ago, and tumour cells became useful tools to evaluate this process, its acceptance as a *bona fide* mechanism of tumour escape required considerable persuasion. Similarly, tumour-mediated immune suppression remains a controversial issue among tumour immunologists, although modern molecular immunology has identified at least a dozen mechanisms attributed to tumours and responsible for immune cell dysfunction in tumour-bearing hosts (Whiteside, 2002).

More than 25 years ago, reports appeared in the scientific literature describing isolation from supernatants of human tumour cell lines of membraneous vesicles (MV) expressing molecular markers characteristic of tumour plasma membranes (Taylor *et al*, 1983a). In cultures of murine B16 melanoma cells, for example, the isolated MV were found to contain glycoprotein profiles identical to those in the tumour cell membrane (Taylor *et al*, 1988). Careful biochemical analysis of MV isolated from the ascites of patients with ovarian carcinoma showed the presence of placental alkaline phosphatase (PLAP), CD44 and TSG101 protein, all present in the tumour as well (Taylor and Gercel-Taylor, 2004). These and other studies not only confirmed the identity of membrane-associated markers with those on the tumour cell surface but also indicated that an energy-dependent process of ‘shedding’ was responsible for their appearance in cell supernatants as well as sera and other body fluids of patients with cancer (Taylor *et al*, 1988; Taylor and Gercel-Taylor, 2005). From the beginning, the presence of MV, which are estimated to be from 50 to 100 nm in diameter, has been linked to cancer progression, largely because of their abundance in body fluids of cancer patients with advanced disease and their paucity or absence in body fluids of normal individuals. The importance of the shedding phenomenon for tumour progression took some years to appreciate, and recent discoveries of a variety of biologically active molecules, such as a membrane form of Fas ligand (FasL), APO2/TRAIL, CD44 or major histocompatibility complex (MHC) class I antigens, in association with MV provide a reasonable explanation for their involvement in immune suppression. It appears that MV shed from the tumour surface and isolated from tumour cell supernatants or body fluids of cancer patients have profound inhibitory effects on immune cells *in vitro*, including blocking of signalling, proliferation, cytotoxicity and induction of apoptosis (Taylor *et al*, 2003; Kim *et al*, 2004). *In vivo*, the development of tumour-related anergy could be linked to the appearance in the patients' sera of shed tumour membrane fragments (Taylor *et al*, 1983b).

However, tumour cells are not the only source of MV in body fluids, as extracellular shedding is commonly encountered in other cell types, among them activated immune cells. This would argue for the existence in body fluids of a variety of MV, representing different cellular origins. Indeed, quite independently but in parallel to then on-going biochemical and structural characterization of tumour-derived MV, investigators discovered vesicular structures produced by maturing reticulocytes and named them ‘exosomes’ (Johnstone *et al*, 1987). Since their first description in late 1980s, exosomes have steadily occupied attention of biologists, and much information has accumulated in regard to their structure and molecular attributes (Thery *et al*, 2002). Exosomes contain a mix of proteins associated with antigen presentation (MHC and costimulatory molecules and heat shock proteins); microvesicular structure (cytoskeleton-associated tubulin, actin-binding proteins, annexins); exosome targeting (the tetraspanin family, lactadherin and Mac-1); and apoptosis (FasL, APO2/TRAIL).

Exosomes became a focus of particular interest to cancer investigators when Zitvogel and co-workers reported that dendritic cells (DC) generated from peripheral blood of cancer patients produce and release exosomes, which accumulate tumour-rejection antigens and thus can be used as antitumour vaccines (Thery *et al*, 1999). To a casual observer, exosomes and MV seemed to represent similar tumour antigen-enriched structures, which were being isolated from DC or sera, respectively, of patients with cancer by methods involving ultracentrifugation but performed under quite different conditions. However, exosomes were being viewed as convenient vehicles for the delivery of DC-processed tumour antigens to T cells by some investigators (Chaput *et al*, 2003), while others looked at tumour-derived MV as harbingers of T-cell death by virtue of their ability to induce apoptosis of activated T cells mediated by membrane-associated FasL and related molecules (Andreola *et al*, 2002; Kim *et al*, 2004). Convincing evidence was mustered by both sides, and thus one was tempted to conclude that DC and tumour cells shed membranous structures with a distinctly different molecular profile and distinct biologic functions. This was a plausible explanation, but as we now learn, not a correct one. In this issue of the journal, the Taylors, who incidentally, are responsible for MV discovery, convincingly argue that exosomes and MV are one and the same entity based on molecular and biochemical evidence (Taylor and Gercel-Taylor, 2005). These investigators obtained ascites or serum specimens from women with ovarian carcinoma, split these specimens into two aliquots and used them to isolate (1) exosomes by the gradient density centrifugation and (2) MV by the two-step chromatography/centrifugation procedure. Then, exosomes and MV were systematically compared for their protein profiles by SDS–PAGE, protein markers expression by Western immunoblots, and biologic activity in functional assays measuring expression of signalling molecules or DNA fragmentation induced by MV in T lymphocytes. The results are unambiguous: exosomes and MV had identical protein profiles and mediated the same immunoinhibitory activities *in vitro*. These results are significant because they: (a) close the gap between two parallel research fields, placing future investigations of exosomes and MV on the same platform; (b) give credence to the fact that MV/exosomes are involved in immune regulation in cancer and (c) open a way for creative novel approaches to the use of these structures in future therapy of cancer.

While the characterization of MV/exosomes isolated from sera and body fluids of patients with various neoplasias continues, a number of concerns remain. For example, it has not been proven that in tumour-bearing subjects, tumour is the only or the major source of origin for these structures. It could be argued, for example, that activated immune cells are the major contributor. It is also possible that once shed from tumour and/or immune cells, these structures rearrange or coalesce to form ‘hybrid’ vesicles with molecular profiles of various contributing cells. Differences in the molecular composition of MV in patients with the same malignancies could exist, accounting for variability in their biologic activity. The status of MV in patients cured of their disease (NED) is not clear, but it appears that they persist in the circulation long after therapy. Taylor *et al* (1983b) reported that the serum level of MV increases prior to the clinical diagnosis of recurrent disease. Our own data suggest that the FasL content of MV in patients with head and neck cancer may be related to disease activity and prognosis, in that patients with Stage III and IV disease and those with lymph node metastases had MV in the sera with the highest content of the 41kDa FasL (Kim *et al*, 2004).

MV isolated from body fluids of patients with various malignancies are enriched in the membrane-bound molecules able to induce apoptosis in activated immune cells. This provides an explanation for spontaneous apoptosis of T lymphocytes observed in the peripheral circulation of patients with cancer (Kim *et al*, 2004). CD8+ effector T cells are preferentially targeted for apoptosis. Differential sensitivity of CD8+ and CD4+ T cells to MV-induced, Fas-mediated apoptosis could be perhaps explained by an incomplete cleavage of caspase-8 to its active form reported to occur in Th2 cells. Protection from MV-incduced apoptosis by cytokine- or chemokine-mediated activation of phosphatidylinositol-3-kinase (PI3K) in these cells might contribute to their relative resilience.

An important aspect of the MV/exosome biology concerns their general role in immune tolerance. Thus, MV have been isolated from maternal blood and are known to be produced by placental cells (Taylor and Black, 1987). Further, a recent paper by Morelli *et al* (2004) suggests that in mice, blood-borne MHC class I-rich allogeneic exosomes are targeted, internalised and processed by DC in the spleen and other organs. These exosomes are processed by CD8*α*+ DC in a way that induces unresponsiveness of T cells to the presented alloantigens. These experiments introduce a possibility that donor-derived exosomes delivered intravenously could induce donor-specific transplant tolerance. In a different context, Licia Rivoltini reports that melanoma cell line-derived exosomes interfere with DC maturation and with tumour-antigen presentation by these DC to T cells in *in vitro* assays (personal communication). Thus, the role exosomes play in DC-mediated T-cell responses is not yet clear. DC appear to be able to edit exosome processing, with a final outcome directed toward tolerance rather than immune stimulation, depending perhaps on the route of MV administration or on their antigenic mosaic. MV/exosomes may function as messengers for positive or negative intercellular signalling and transfer of molecules between T cells, DC and the tumour or foreign graft. Caution is, therefore, needed in using MV/exosomes as antitumour vaccines for fear of inducing tolerance. Dissection of immunogenic *vs* tolerogenic signals delivered by these structures to DC or T lymphocytes is an important future challenge. There is evidence that MV can directly downregulate expression of the TcR-associated *ζ* chain as well as expression of JAK3 in Jurkat cells co-incubated with MV. While these data confirm suppressive effects of MV on activated T cells, a better understanding of the molecular mechanisms and signalling pathways targeted by MV/exosomes are necessary.

MV/exosomes are a perfect example of the efficiency of tumours in orchestrating their escape from the host immune system. By adapting a ubiquitous process of membrane shedding, tumours are able to accomplish the loss of those antigens that may be immunogenic and capable of signalling to immune cells as well as induce dysfunction or death of immune effector cells located at a distance from the tumour microenvironment. A better understanding of this process, and especially of molecular mechanisms involved in exosome/MV interactions with immune cells, is likely to provide significant insights into the phenomenon of tumour-related immune tolerance. In addition, insights into the MV/exosome biology are likely to offer opportunities for the development of novel approaches to immunotherapy of cancer, graft rejection and other diseases.
